# Increasing Newly Diagnosed Rate and Changing Risk Factors of HCV in Yanbian Prefecture, a High Endemic Area in China

**DOI:** 10.1371/journal.pone.0086190

**Published:** 2014-01-27

**Authors:** Hong-Xin Piao, Ai-Ting Yang, Ya-Meng Sun, Yuan-Yuan Kong, Xiao-Ning Wu, Ying-Zhe Zhang, Bo Ding, Bao-En Wang, Ji-Dong Jia, Hong You

**Affiliations:** 1 Liver Research Center, Beijing Friendship Hospital, Capital Medical University, Beijing, China; 2 Affiliated Hospital of Yanbian University, Yanji, Jilin, China; University of New South Wales, Australia

## Abstract

**Background:**

The newly diagnosed rate of HCV infection is increasing in China. However, the risk factors have not been fully identified. Here, a survey was performed in Yanbian Prefecture, a high-endemic area in China.

**Methods:**

We identified newly diagnosed HCV infection in 2007–2011, using the local National Disease Supervision Information Management System from the Chinese Center for Disease Control and Prevention. We determined the risk factors using a case-control survey by questionnaire.

**Results:**

Yanbian Prefecture had a rapid increase in the yearly newly diagnosed rate of HCV infection from 32.6 to 72.1/100.000 from the year 2007 to 2011. People aged 50–64 years had a high HCV infection of 43.4%, but only 0.3% of cases were reported in those aged less than 20 years. Cosmetic treatment, family history, blood transfusion, and dental treatment were independent risk factors for HCV infection. Unexpectedly, cosmetic treatments [odd ratio (OR) = 5.15, 95% confidence interval (CI) = 2.31–11.48, *P* = 0.00] and family history (OR = 4.68, 95% CI = 2.67–8.75, *P* = 0.00) showed a higher risk than the conventional risk factors of blood transfusion (OR = 4.49, 95% CI = 1.95–10.37, *P* = 0.001) and dental treatment (OR = 2.98, 95% CI = 1.42–6.25, *P* = 0.00). To further analyze the intrafamilial transmission, we found that spouses of HCV patients had an increased risk for acquiring HCV (OR = 5.75, 95% CI: 1.94–17.07), without significant association between either HCV RNA viral load (*P* = 0.29) or genotype (*P* = 0.43).

**Conclusions:**

HCV infection was increased in Yanbian Prefecture. Cosmetic treatment was a higher risk factor than medical procedure. HCV infection had a clear family clustering phenomenon, especially between spouses.

## Introduction

HCV infection is a major public health problem worldwide. There are about 170 million patients with HCV and 3–4 million new cases are diagnosed every year [1]–[3].

Recently in China, a survey showed that total HCV infection rate was 0.4% in the human population in six regions of Beijing, Heilongjiang, Shandong, Ningxia, Gansu and Sichuan in 2006–2008 [4]. In addition, positive rate of HCV varies throughout China with significant regional differences, for example Heilongjiang province (Northeast of China) has a higher infection rate (0.74%) than other regions [5]–[6].

Yanbian Korean Autonomous Prefecture (hereafter referred to as Yanbian Prefecture) is also located in the high-endemic area of Jilin Province (Northeast of China). However, no data have been reported for the newly diagnosed rate and risk factors of HCV infection in Yanbian Prefecture. According to a study of 690 patients from around Anyang in China in 2009, the top three risk factors for HCV infection were intravenous use of glass syringes/needles (75.4%), blood transfusion (73.9%) and oesophageal balloons (27.5%) [7]. However, the epidemiology of HCV transmission in a high-prevalence area is less well understood, including Yanbian Prefecture.

In this study, we evaluated the reported newly diagnosed rate of HCV and explored possible risk factors for acquiring the infection. We hope that the findings might guide the development, adaptation, and evaluation of prevention strategies.

## Materials and Methods

### Study design

This study was conducted in two stages. The first stage was a retrospective study to investigate the newly diagnosed rate trends of HCV in Yanbian Prefecture from 2007 to 2011, and the second stage was a hospital-based case-control study to identify the risk factors for HCV infection and non-HCV infection.

For study population I, we collected data on episodes of newly diagnosed HCV infection from January 2007 to December 2011, using the National Disease Supervision Information Management System from the China Information System for Disease Control and Prevention (CDC). To calculate the newly diagnosed rate of HCV infection, we collected demographic data from the Bureau of Statistics of Yanbian Prefecture.

For study population II of hospital-based cases, to identify the risk factors, we performed a 1∶1 matched case-control study. We screened 200 HCV antibody positive and HCV RNA positive inpatients and outpatients from January to December 2009. For each positive case, one HCV-negative age- and sex-matched patient was enrolled as a control. The cases sample was selected from the HCV antibody positive and HCV RNA positive patients by a systematic sampling procedure.

The study protocol was approved by the Ethics Committee of Capital Medical University in accordance with the Declaration of Helsinki. Written informed consent for participation in the study was obtained from each patient.

### Family members

Family members were defined as parents, spouses, brothers/sisters, and relatives living close together. HCV infected family members were identified through medical record. In all, 49 family members of HCV cases and 19 family members of controls were enrolled. All family members of HCV infected were tested for HCV RNA and genotype.

### Questionnaire

We designed a questionnaire on risk factors for HCV infection based on a case- control investigation questionnaire on HCV infection in Yanbian Prefecture, designed by the Chinese CDC national immunization program in 2009, and risk factors reported in the literature. This questionnaire focused on sociodemographic information, medical behaviour, community and sexual behaviour, daily life behaviour and family member history.

### Laboratory methods

Venous blood (4 mL) samples were refrigerated and transported from the collection site to the central laboratory at an Affiliated Hospital of Yanbian University every second day. Blood samples were screened for HBsAg, anti-HBc IgM, anti-HCV, anti-HAV IgM, by a third-generation enzyme immunoassay (Kehua Bio-engineering Co. Ltd, Shanghai, China). Qualitative detection of serum HCV RNA was performed by quantitative real-time PCR (PG Biotech Company, Shenzhen, China) according to the manufacturer's instructions. Genotype was determined by RT-PCR using type specific primers (Sansure Biotech Inc., Hunan, China) and classified according to the manufacturer's instructions.

### Statistical analysis

The data were entered (double entry) and analyzed using SPSS for Windows version 16.0. Comparisons between groups were made by the χ^2^ or Fisher's exact test for categorical variables, and by the *t* test and ANOVA test for quantitative variables. The odds ratio (OR) and 95% confidence interval (CI) for the factors under consideration were calculated in a univariate and multivariate logistic regression analysis. *P*<0.05 was considered to be statistically significant.

## Results

### Increased newly diagnosed rate of HCV infection in Yanbian Prefecture

**Figure 1:**
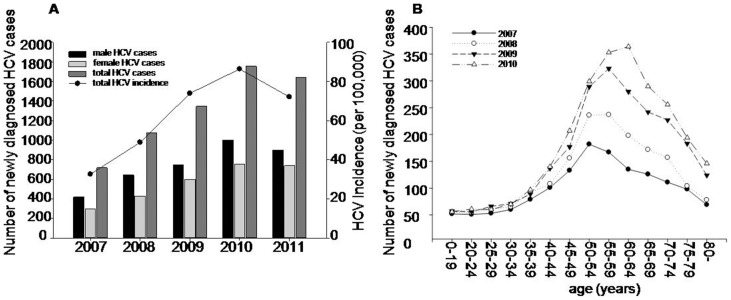
HCV infection was related to sex and age. A. Number of female, male, total cases and total newly diagnosed rate of newly diagnosed from 2007 to 2011 in Yanbian Prefecture, China. **B.** Number of HCV cases by age from 2007 to 2011.

During 2007–2011, 6531 new cases of HCV infection were diagnosed, and the average newly diagnosed rate was 62.8/100,000 in Yanbian Prefecture. The newly diagnosed rate was lowest (32.6/100,000) in 2007 and then increased by 39.3% per year on average. The highest HCV newly diagnosed rate (86.3/100,000) was in 2010, and then slightly decreased to 72.1/100,000 in 2011. With respect to sex, the average newly diagnosed rate was higher in males (67.3/100,000) than in females (46.8/100,000) (ratio 1.47∶1, χ^2^ = 40.99, *P*<0.01). (Figure 1A)

Between 2007 and 2011, only 20 cases (0.3%) were reported in persons under age 20 years, while most cases were in those aged elder than 20 years. The age-specific newly diagnosed rate evaluated in 5-year intervals increased steeply from age 30–34 years old. The peak age shifted from 50–54 in the year of 2007 to 60–65 in the year of 2010. Notably, the main cases (75.8%) were recorded in the 50–79 years age groups. (Figure 1B)

### Demographic characteristics among HCV cases and non-HCV controls

To identify the risk factors of HCV infection in Yanbian Prefecture, we conducted a case-control study with 200 HCV cases and 200 non-HCV cases as controls. No significant differences were found between the demographic and socioeconomic characteristics of the cases and controls. These results are summarised in Table S1. Of the 200 HCV cases, 52.5% were males, the most frequent age group was 41–60 years (60.0%) and most marital status were married (97.0%) as well as the general population.

### Risk factors for HCV infection

We evaluated numbers of risk factors, including those associated with medical, community and sexual and daily life behaviour. As expected, nearly all risk factors associated with medical behaviour were positively associated with hepatitis C using the non-HCV as controls. The following were significant risk factors: history of surgery (49.0% *vs.* 33.5%, *P* = 0.00), dialysis (7.0% *vs.* 2.5%, *P* = 0.03), blood transfusion (29.0% *vs.* 6.0%, *P* = 0.00), acupuncture (5.5% *vs.* 1.5%, *P* = 0.03), dental treatment (82.0% *vs.* 57.0%, *P* = 0.00), endoscopy (42.0% *vs.* 21.5%, *P* = 0.00), sharing needles (6% *vs.* 1%, *P* = 0.01), and intravenous/intramuscular injection (81.0% *vs.* 68.5%, *P* = 0.00), while there was no association with history of blood donation (6% *vs.* 10.5%, *P* = 0.10). These results indicated that invasive medical procedures were still the main route of HCV transmission in Yanbian Prefecture (Table 1).

**Table 1:** Risk factors related to medical behaviour among HCV and non-HCV cases.

Variable	HCV	non-HCV	OR	95% CI	*P*
	n = 200 (%)	n = 200 (%)			
Surgery			1.91	1.12–2.86	0.00
No	102 (51.0)	133 (66.5)			
Yes	98 (49.0)	67 (33.5)			
Dialysis			2.94	1.04–8.31	0.03
No	186 (93.0)	195 (97.5)			
Yes	14 (7.0)	5 (2.5)			
Blood transfusion			6.40	3.31–12.36	0.00
No	142 (71.0)	188 (94.0)			
Yes	58 (29.0)	12 (6.0)			
Blood transfusion			0.54	0.26–1.14	0.10
No	188(94.0)	179 (89.5)			
Yes	12 (6.0)	21 (10.5)			
Acupuncture			3.88	1.05–13.91	0.03
No	189 (94.5)	197 (98.5)			
Yes	11 (5.5)	3 (1.5)			
Dental treatment			3.44	2.18–5.43	0.00
No	36 (18.0)	86 (43.0)			
Yes	164 (82.0)	114 (57.0)			
Endoscopy			2.64	1.71–4.10	0.00
No	116 (58.0)	157 (78.5)			
Yes	84 (42.0)	43 (21.5)			
Sharing needle			6.32	1.40–28.61	0.01
No	188 (94.0)	198 (99.0)			
Yes	12 (6.0)	2 (1.0)			
Intravenous/intramuscular injection			1.96	1.24–3.11	0.00
No	38 (19.0)	63 (31.5)			
Yes	162 (81.0)	137 (68.5)			

With respect to risk factors associated with community and sexual behaviour (Table 2), risk for HCV infection was higher in patients with cosmetic treatment (38.0% *vs.* 19.0%, *P* = 0.00) and those shaved at the barber (46.0% *vs.* 15.0%, *P* = 0.00), without close relation with bathing at public bathroom, numbers of sexual partners and using marijuana. This may indicate that mucous membrane exposure was another risk factor for HCV infection in Yanbian Prefecture. We also noted that using condoms was a protective factor against HCV infection (8.0% *vs.* 33.5%, *P* = 0.00).

**Table 2:** Risk factors related to community and sexual behaviour among HCV and non-HCV cases.

Variable	HCV	non-HCV	OR	95% CI	*P*
	n = 200 (%)	n = 200 (%)			
Cosmetic treatment			2.61	1.66–4.11	0.00
No	124 (62.0)	162 (81.0)			
Yes	76 (38.0)	38 (19.0)			
Bathing at public bathroom			1.51	0.72–3.15	0.27
No	13 (6.5)	19 (9.5)			
Yes	187 (93.5)	181 (90.5)			
Shaving at barber			4.83	2.30–7.78	0.00
No	108 (54.0)	170 (85.0)			
Yes	92 (46.0)	30 (15.0)			
Using condom			0.17	0. 10–0.31	0.00
No	184 (92.0)	133 (66.5)			
Yes	16 (8.0)	67 (33.5)			
Number of sexual partners			1.13	0.43–2.30	0.80
Single	191 (95.5)	192 (96.0)			
Multiple	9 (4.5)	8 (4.0)			
Using marijuana			6.16	0.73–51.60	0.12
No	194 (97.0)	199 (99.5)			
Yes	6 (3.0)	1 (0.5)			

Consistent with previous reports, our study showed that factors associated with daily life behaviour, including sharing toothbrushes, razors, nail clippers and bath towels, did not differ between cases and controls. However, interestingly, household contact with HCV carriers was more frequent in HCV cases than in controls (24.5% *vs.* 9.5%, *P* = 0.00) (Table 3).

**Table 3:** Risk factors related to daily life behaviour among HCV and non-HCV cases.

Variable	HCV	non-HCV	OR	95% CI	*P*
	n = 200 (%)	n = 200 (%)			
Household contact with HCV carrier			3.09	1.75–5.49	0.00
No	151 (75.5)	181 (90.5)			
Yes	49 (24.5)	19 (9.5)			
Mother with HCV			1.88	0.68–5.19	0.22
No	189 (94.5)	194 (97.0)			
Yes	11 (5.5)	6 (3.0)			
Father with HCV			1.42	0.44–4.53	0.56
No	193 (96.5)	195 (97.5)			
Yes	7 (3.5)	5 (2.5)			
Spouse with HCV			5.75	1.94–17.07	0.00
No	179 (89.5)	196 (98.0)			
Yes	21 (10.5)	4 (2.0)			
Brother/sister with HCV			2.38	0.61–9.36	0.20
No	193 (96.5)	197 (98.5)			
Yes	7 (3.5)	3 (1.5)			
other family members with HCV			3.03	0.31–29.38	0.62
No	197 (98.5)	199 (99.5)			
Yes	3 (1.5)	1 (0.5)			
Toothbrushes sharing			0.75	0.17–3.38	1.00
No	197 (98.5)	196 (98.0)			
Yes	3 (1.5)	4 (2.0)			
Razor sharing			1.39	0.62–3.11	0.42
No	185 (92.5)	189 (94.5)			
Yes	15 (7.5)	11 (5.5)			
Nail clipper sharing			1.07	0.71–1.62	0.75
No	67 (33.5)	70 (35.0)			
Yes	133 (66.5)	130 (65.0)			
Bath towel sharing			0.85	0.54–1.34	0.49
No	154 (77.0)	148 (74.0)			
Yes	46 (23.0)	52 (26.0)			

### Family clustering of HCV infection mainly though spouses

As shown in Table 3, we found that the proportion of spouses with HCV in cases group (10.5%) was significantly higher than that of control group (2.0%, *P* = 0.00). It indicated intrafamilial transmission of HCV infection may be predominant between spouses.

In non-HCV group, the proportion of mother (3%) with HCV was higher than father (2.5%), brother/sister (1.5%) and other family members (0.5%); in HCV group, the proportion of mother (5.5%) with HCV was also higher than father (3.5%), brother/sister (3.5%) and other family members (1.5%). Although the proportion of mother with HCV was higher than other relatives, no significant difference was found (*P* = 0.22).

To evaluate whether HCV RNA or genotype associated with intrafamilial transmission, we analyzed all of the identified HCV patients in two groups. There were 49 cases in HCV group and 19 cases in non-HCV group. As shown in Table 4, we found that there was no significant difference in HCV RNA between HCV and non-HCV group (6.1 IU/mL *vs.* 6.5 log IU/mL, *P* = 0.29). In additional, family members with HCV, genotype 2a in HCV group (42.9%) was higher than that of non-HCV group (26.3%), but there were no significantly statistical difference between them (*P* = 0.43).

**Table 4:** Virology of family members with HCV within HCV and non-HCV group.

Variable		family member with HCV within HCV group							family member with HCV within non-HCV group							*P*
		n = 49							n = 19							
		Mother	Farther	Spouse	Brother/sister	other family members	*P*	total	Mother	Farther	Spouse	Brother/sister	other family members	*P*	total	
		N = 11	N = 7	N = 21	N = 7	N = 3			N = 6	N = 5	N = 4	N = 3	N = 1			
HCV RNA	Medium	6.6	6.3	6.4	5.9	5.8	0.62	6.1	6.3	6.5	6.1	7.7	5.4	0.63	6.5	0.29
(log IU/mL)	Range	5.5–7.1	4.4–6.4	5.6–6.9	4.9–6.2	4.5–6.7		5.3–6.8	5.7–6.7	6.1–7.2	5.3–7.1	4.5–7.8	5.4–5.4		5.4–7.0	
Genotype	1b	5	3	13	1	1	0.24	23	3	2	3	3	1	0.32	12	0.43
	(%)	(45.5)	(42.9)	(61.9)	(14.3)	(33.3)		(46.9)	(50.0)	(40.0)	(75.0)	(100)	(100)		(63.2)	
	2a	6	2	6	6	1		21	1	3	1	0	0		5	
	(%)	(54.5)	(28.6)	(28.6)	(85.7)	(33.3)		(42.9)	(16.7)	(60.0)	(25.0)	(0)	(0)		(26.3)	
	Unknown	0	2	2	0	1		5	2	0	0	0	0		2	
	(%)	(0)	(28.6)	(9.5)	(0)	(33.3)		(10.2)	(33.3)	(0)	(0)	(0)	(0)		(10.5)	

Finally, we analyzed the genotypes consistency of the cases and the family members. There were 66.7% spouses had the same genotype, higher than 16.7% among sisters/brothers, which indicated that intrafamilial transmission might be predominant through sexual pathway. However, since the limited number of available cases, it should be cautious to interpret the data (Table S2).

### Multivariate analysis findings

**Figure 2:**
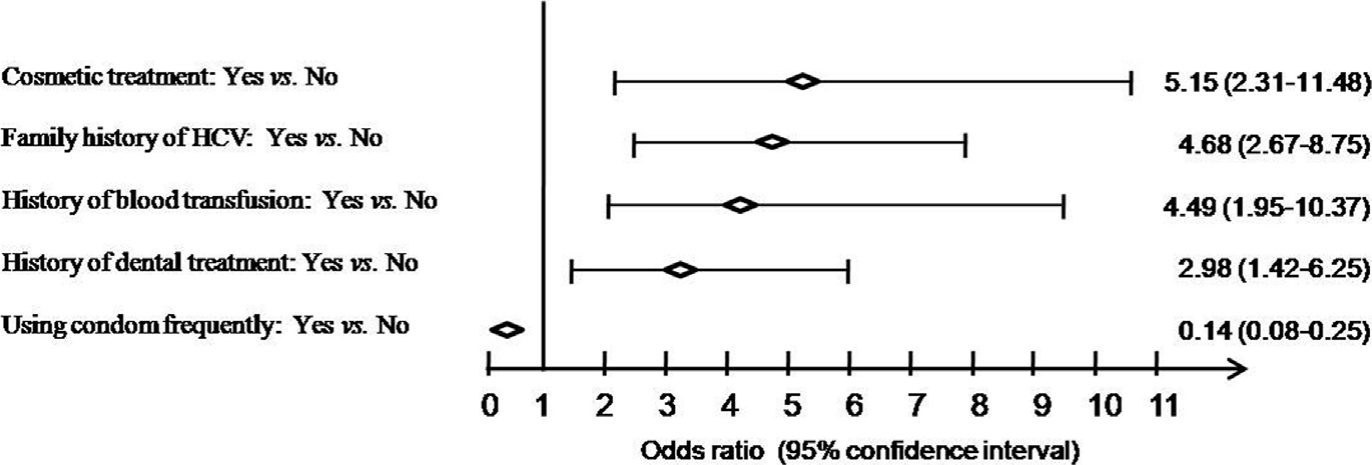
Odds ratio and 95% confidence interval was shown for the independent HCV risk factors by multivariate analysis. Cosmetic treatment, family history of HCV, history of blood transfusion, and history of dental treatment were risk factors, whereas using condom was a protective factor.

In our multivariate model, conventional factors such as a history of dental treatment (OR = 2.98, 95% CI = 1.42–6.25, *P* = 0.00) and blood transfusion (OR = 4.49, 95% CI = 1.95–10.37, *P* = 0.00) were independently associated with HCV infection. Using condom was a protective factor (OR = 0.14, 95% CI = 0.08–0.25, *P* = 0.00). Interestingly, a history of cosmetic treatment was the most strongly related to the risk of HCV infection (OR = 5.15, 95% CI = 2.31–11.48, *P* = 0.00), and a family history of HCV was also a high risk factor (OR = 4.68, 95% CI = 2.67–8.75, *P* = 0.00) (Figure 2).

## Discussion

With epidemiological analysis based on Chinese CDC data, we showed that the newly diagnosed rate of Yanbian Prefecture had risen sharply since 2007–2010. The average newly acquired HCV rate (62.8/100,000) in Yanbian Prefecture was significantly higher than the national average (8.21/100,000) and that for blood donors (24.2/100,000) [8]–[9]. It is worth nothing that the newly diagnosed rate of HCV in Yanbian Prefecture, not referred the incidence, may not accurately reflect the newly acquired HCV. Since people can be infected with HCV for many years without diagnosis, in addition to lacking previous recent negative HCV evidence, it should be caution to interpret the trend.

When comparing the high prevalence areas of HCV, there are differences of ages and risk factors.

We found that HCV newly diagnosed rate was significantly higher in older individuals (more than 50 years of age), who accounted for three-quarters of all infections in Yanbian Prefecture, parallels data found in cohorts from Taiwan and Japan [10]–[12]. It is plausible that 40–60 years population has more chance to exposure to sex, cosmetic treatment, HCV carrier, blood transfusion, and dental treatment than other ages. In contrast, in the United Kingdom between 1996 and 2008, half of all received reports were among individuals aged between 25 and 39 years, as well as in Australia and American. This may be induced by the high drug user proportion in those countries [13]–[15].

Among risk factors of HCV, besides the common known ones such as invasive medical procedures and blood transfusion, our study identified two major risk factors including cosmetic treatment or close contacting with HCV carrier/patients, especially those spouses among family members.

Beauty or cosmetic treatment is becoming major risk factor of hepatitis C in some high prevalence areas, although it is known that different risk factors are predominant in other parts of the world. For example, in Pakistan, shaving of beards has been identified as a route of acquiring HCV infection [16]. In France, Karmochkine *et al.* [17] have also demonstrated that undergoing beauty or cosmetic treatment or having a professional pedicure/manicure is significantly associated with HCV. In China, it has also been reported that razor sharing and ear piercing are risk factors in Chengdu [18]. It can be seen that transmission routes of HCV infection are changing continuously.

Family clustering continues to be another important but controversial risk factor in HCV epidemiology. In our study, we confirmed that contact with an HCV carrier/patient in the family was a risk factor for infection. Intrafamilial transmission of HCV infection was mainly through sexual activity between spouses. Recently, there have been reports of the presence of the hepatitis C virus RNA was found in the semen of up to 1/3 of HCV viremic men [19]. That could partially explain that why using condom was a protective factor against HCV infection in our study. To further evaluate risk factors (HCV RNA and genotypes) associated with intrafamilial transmission of HCV unfortunately, there was no statistical significant relationship with HCV RNA or genotype. Although genotype 2a was more common in family members in HCV group than non-HCV group, the difference between them was not significant (*P* = 0.45).

Similarly, Magder *et al.* [20] have reported that HCV is transmitted between spouses in Egypt. Based on the models, 40 (6%) of 694 married individuals with anti-HCV antibodies in the two communities diagnosed HCV infection from their spouses. A systematic review has also shown that intrafamilial transmission of HCV does occur [21], especially between offspring (OR = 1.77), siblings (OR = 9.75), and spouses (OR = 20.57). However, previous studies have reported that the spread of HCV infection among family members of patients with HCV infection is uncommon. In Korea, Kim *et al.* [22] have tested HCV infection among 250 family members (104 men, 146 women) with HCV infection and 170 family members of control groups (64 men, 106 women). They found that only one family member of the HCV cases and no one related to the controls had HCV infection. Hajiani *et al* [23] have reached a similar conclusion: HCV seroprevalence among the household contacts of HCV carriers was 1.3%, which was not significantly higher than that in the controls (1.0%, *P*>0.05).

Although injecting drug use had the strong trend with HCV (OR = 6.16, 95% CI = 0.73–51.60), compared with non injecting drug use, the low amount (7/400) of drug-users did not had the power showing statistical significance (*p* = 0.12). The reasons for this may be drug users relatively rare in Yanbian Prefecture and under reporting. Since the history of injecting drug use collected by self-reporting questionnaire, it cannot rule out patients concealing some information.

When we found the changing risk factors in the high prevalence of HCV area, we also notice that there are several limitations of our study. First, although the incidence of HCV was conducted in a large population-based survey, only 200 HCV patients and 200 non-HCV patients were enrolled to evaluate the risk factors. The small sample size may have resulted in insufficient power to detect all of the risk factors associations between diagnosed HCV. Second, our analysis was performed retrospectively. Ideally, prospective cohort studies would be the most adequate approach to obtain valid estimates of the association between HCV acquisition and potential risk factors. Third, to explore the mechanisms of intrafamilial transmission, we should detect interleukin-28 single nucleotide polymorphism, or other immune markers which indicate that differentiates susceptible persons contacted with HCV carriers. Especially, phylogenetic analysis would be ideal to assess the transmission among family members. Those experiments should been done to identify the potential mechanisms.

In summary, we found that the newly diagnosed rate of HCV infection was as high as 62.8/100,000 in Yanbian Prefecture. The case-control study identified several risk factors for HCV infection. Interestingly, cosmetic treatment and intrafamilial spread underscores the importance of medical procedures (history of blood transfusion and dental treatment) in HCV transmission.

## Supporting Information

Table S1 **Distribution of demographic and socioeconomic characteristics of 200 HCV and 200 non-HCV cases.** No significant differences were found between the demographic and socioeconomic characteristics of the cases and controls.
(DOCX)

Table S2 **Genotypes of the case and the family member within HCV group.** There were 66.7% spouses had the same genotype, higher than 16.7% among sisters/brothers, which indicated that intrafamilial transmission might be predominant through sexual pathway.
(DOCX)
