# Supplementary material for: Increasing Newly Diagnosed Rate and Changing Risk Factors of HCV in Yanbian Prefecture, a High Endemic Area in China
Source: PLoS One. 2014 Jan 27;9(1):e86190. doi: 10.1371/journal.pone.0086190 (PMC3903515; doi:10.1371/journal.pone.0086190)
Supplement: Table S1 — Distribution of demographic and socioeconomic characteristics of 200 HCV and 200 non-HCV cases. No significant differences were found between the demographic and socioeconomic characteristics of the cases and controls. (DOCX) [file pone.0086190.s001.docx]

**Table S1.**

| **Variable** | **HCV** | **non-HCV** | **χ^2^** | ***P*** |
| --- | --- | --- | --- | --- |
|  | **N = 200 (%)** | **N = 200 (%)** |  |  |
| Sex |  |  | 0.00 | 1.00 |
| Male | 105 (52.5) | 105 (52.5) |  |  |
| Female | 95 (47.5) | 95 (47.5) |  |  |
| Age (yr) |  |  | 1.85 | 0.76 |
| 1-20 | 3 (1.5) | 4 (2.0) |  |  |
| 21-40 | 19 (9.5) | 18 (9.0) |  |  |
| 41-60 | 120 (60.0) | 117 (58.5) |  |  |
| 61-80 | 55 (27.5) | 60 (30.0) |  |  |
| > 81 | 3 (1.5) | 1 (0.5) |  |  |
| Local residence time (yr) |  |  | 0.4 | 0.98 |
| < 1 | 4 (2.0) | 4 (2.0) |  |  |
| 1-5 | 14 (7.0) | 13 (6.5) |  |  |
| > 5 | 182 (91.0) | 183 (91.5) |  |  |
| Marital status |  |  | 0.41 | 0.52 |
| Single | 6 (3.0) | 4 (2.0) |  |  |
| Married | 194 (97.0) | 196 (98.0) |  |  |
| Monthly income (yuan) |  |  | 1.05 | 0.59 |
| < 1000 | 85 (42.5) | 87 (43.5) |  |  |
| 1000-4000 | 109 (54.5) | 110 (55.0) |  |  |
| > 4000 | 6 (3.0) | 3 (1.5) |  |  |
