# Supplementary material for: Increasing Newly Diagnosed Rate and Changing Risk Factors of HCV in Yanbian Prefecture, a High Endemic Area in China
Source: PLoS One. 2014 Jan 27;9(1):e86190. doi: 10.1371/journal.pone.0086190 (PMC3903515; doi:10.1371/journal.pone.0086190)
Supplement: Table S2 — Genotypes of the case and the family member within HCV group. There were 66.7% spouses had the same genotype, higher than 16.7% among sisters/brothers, which indicated that intrafamilial transmission might be predominant through sexual pathway. (DOCX) [file pone.0086190.s002.docx]

**Table S2.**

| **Genotype** | **Family member with HCV (n = 49)** | | | |
| --- | --- | --- | --- | --- |
|  | Parent (n = 18) | Spouse (n = 21) | Sister/ brother (n = 7) | Others (n = 3) |
| Detected | 1 | 18 | 6 | 3 |
| Same | 0 (0) | 12 (66.7%) | 1 (16.7%) | 1 (33.3%) |
| Different | 1 (100%) | 6 (33.3%) | 5 (83.3%) | 2 (66.7%) |
